# Probing fluorination promoted sodiophilic sites with model systems of F_16_CuPc and CuPc

**DOI:** 10.1007/s12200-022-00026-3

**Published:** 2022-04-28

**Authors:** Yuan Liu, Xu Lian, Zhangdi Xie, Jinlin Yang, Yishui Ding, Wei Chen

**Affiliations:** 1 Joint School of National University of Singapore and Tianjin University, International Campus of Tianjin University, Binhai New City, Fuzhou, 350207 China; 2 Department of Chemistry, National University of Singapore, 3 Science Drive 3, Singapore, 117543 Singapore; 3 Centre for Advanced 2D Materials, National University of Singapore, 6 Science Drive 2, Singapore, 117546 Singapore; 4 Department of Physics, National University of Singapore, 2 Science Drive 3, Singapore, 117542 Singapore

**Keywords:** Fluorination, Phthalocyanines, Sodium metal anode, Sodiophilic sites, In-situ X-ray photoelectron spectroscopy (XPS)

## Abstract

Sodium metal batteries (SMBs) are receiving broad attention due to the high specific capacity of sodium metal anodes and the material abundance on earth. However, the growth of dendrites results in poor battery performance and severe safety problems, inhibiting the commercial application of SMBs. To stabilize sodium metal anodes, various methods have been developed to optimize the solid electrolyte interphase (SEI) layer and adjust the electroplating/stripping behavior of sodium. Among the methods, developing anode host materials and adding electrolyte additives to build a protective layer are promising and convenient. However, the understanding of the interaction process between sodium metal and those organic materials is still limited, but is essential for the rational design of advanced anode hosts and electrolyte additives. In this study, we use copper(II) hexadecafluorophthalocyanine (F_16_CuPc), and copper(II) phthalocyanine (CuPc), as model systems to unravel the sodium interaction with polar functional groups by in-situ photoelectron spectroscopy and density functional theory (DFT) calculations. It is found that sodium atoms prefer to interact with the inner pyrrolic nitrogen sites of CuPc, while they prefer to interact with the outer aza bridge nitrogen atoms, owing to Na-F interaction at the Na/F_16_CuPc interface. Besides, for the both organic molecules, the central Cu(II) ions are reduced to Cu(I) ions by charge transfer from deposited sodium. The fluorine-containing groups are proven to promote the interaction process of sodium in organic materials, which sheds light on the design of functional interfaces in host materials and anode protective layers for sodium metal anodes.

**Graphical Abstract:**

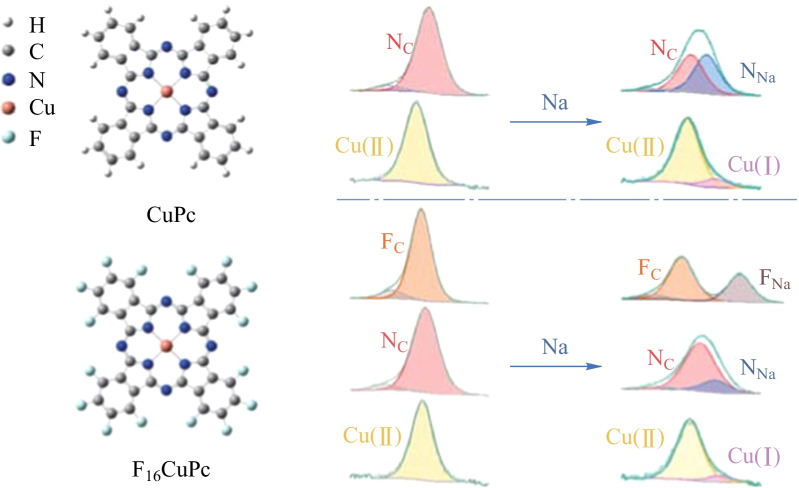

**Supplementary Information:**

The online version contains supplementary material available at 10.1007/s12200-022-00026-3.

## Introduction

Since the development of continuous industrialization and increasing energy demand, sodium metal batteries (SMBs) have attracted extensive attention because of their high theoretical capacity (1166 mAh/g), low redox potential (− 2.71 V vs. SHE), high natural material abundance, and low cost [1]. Nevertheless, many problems hinder their practical application and commercialization, including uncontrollable sodium dendrite growth, poor cycling performance, low coulombic efficiency, and huge volume fluctuation [2, 3]. Among these, the major issue on sodium metal anode is the uneven sodium metal deposition during the operation of the battery, leading to uncontrolled sodium dendrite growth, cell shorting, and severe safety issues. Another problem is the high reactivity of sodium metal with organic electrolytes, generating a fragile solid electrolyte interphase (SEI), which cannot withstand massive volume expansion during cycling, exacerbating the formation of SEI crack, and leading to low coulombic efficiency. To achieve stable sodium metal anodes in a liquid electrolyte, many strategies have been developed [2–6]: (1) electrolyte formulation optimization, including using an ether-based electrolyte, adding additives to adjust the SEI formation, and adjusting the electrolyte concentration; (2) introducing a protective layer to separate bulk sodium metal and electrolyte, and guide uniform sodium deposition by regulating ion flow; (3) building sodium deposition host to reduce local current density, and relieve significant volume expansion during cycling.

Electrolyte formulation optimization is of great importance to improve the properties of the SEI layer for sodium batteries, especially with highly reactive sodium metal anodes, since SEI components mainly come from the decomposition of electrolyte species [4]. Among the various methods, like using ether-based electrolyte [7–9], adding electrolyte additives [10–12], and adjusting electrolyte concentration [13, 14], adding electrolyte additives is a simple but effective promising strategy to stabilize SEI, and improve the cycling performance of SMBs. Based on film-forming and ion-plating strategies, many additives have been developed, including fluoroethylene carbonate (FEC) [10, 15], sodium polysulfide (Na_2_S_6_) [11], and potassium bis(trifluoromethylsulfonyl)imide (KTFSI) [12]. They are all proven to contribute to the formation of a robust SEI layer and regulate the ion-plating manner with suppressed dendrite growth. Introducing protective layers such as artificial SEIs before assembling the batteries is another effective strategy to stabilize sodium metal anodes, since mostly in-situ formed SEI is unstable during prolonged cycling. With an artificial SEI, direct contact between the liquid electrolyte and sodium metal anode can be prevented. Moreover, the sodium ion flux can be regulated and the tremendous mechanical strength can help inhibit the dendrite formation. To construct artificial SEIs on sodium metal, strategies can be divided into chemical pretreatment [16, 17], and thin films deposition by physical technologies [18–20]. Based on this, many useful protective layers have been successfully developed like NaI [16], sodium benzenedithiolate (PhS_2_Na_2_) [17], ion-rich polymeric membrane [21], and poly (vinylidene fluoride) (PVDF)-based layer [22]. In addition, building a sodium deposition host is a highly effective way to mitigate the volume fluctuation of sodium anode and guide homogeneous sodium deposition [23]. With a large specific surface area as well as high electroconductivity, carbon-based materials have been widely studied as advanced skeletons to reduce local current density, alleviate giant volume expansion, and promote uniform sodium deposition, including graphene [24–27], carbon nanotubes [28, 29], and carbon fibers [30–33]. Furthermore, heteroatom doping is a widely used and effective way to introduce “sodiophilic” sites, thereby reducing sodium nucleation barrier and inducing uniform sodium deposition. Based on this, many attractive hosts have been successfully developed, like B-doped graphene (BG) [26], S/N-doped carbon fibers (D-HCF) [32] and O/N-doped carbon nanofibers (ONCNFs) [33].

In general, in order to realize the wide commercial application of sodium metal anode, utilization of electrolyte additives, construction of protective layers and sodium deposition hosts have been widely investigated. Although many achievements have been made, due to the complex electrolyte system in real battery systems, the understanding of interfacial processes and components of SEI is still limited. There are very few systematic investigations on the role of organic additives containing different functional groups during sodium deposition. At present, researches on organic electrolyte additives mostly lie in lithium metal batteries (LMBs). Application and research related to SMBs are rare, and their design mostly imitates the additives in LMBs. However, in different battery systems, the same electrolyte additives could show different or even opposite effects. For example, Wang et al. reported that Na_2_S_6_ alone is beneficial to achieve long-term stability and reversibility, while Na_2_S_6_–NaNO_3_ co-additive has an adverse effect, which contrasts to the previous study in the lithium anode system [11, 34]. Therefore, it is necessary to comprehensively study the roles of electrolyte additives in SMBs. Additionally, various sodium deposition hosts show different properties to inhibit dendrite growth, and improve dendrite growth. Therefore, it is of great importance to study in depth the “sodiophilic” sites in different hosts to establish the structure–function relationship for the rational design of the host framework.

**Fig. 1:**
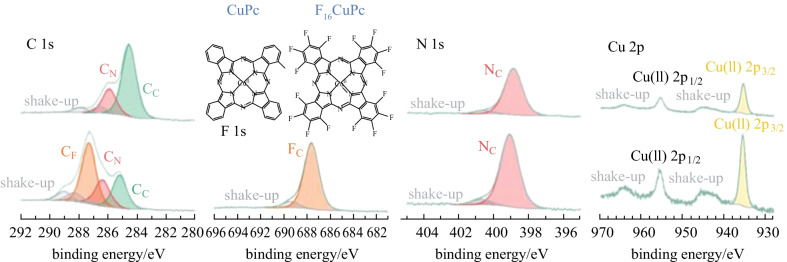
CuPc and F_16_CuPc molecular structures and their characteristic XPS spectra (silicon foils as substrates)

Phthalocyanines (Pcs) and their derivates, with diverse structure and unique charge centers are promising and functional in various batteries [35], such as serving as electrodes in metal-ion batteries [36–38], catalytic additives in Li–S batteries [39, 40], and metal-air batteries [41, 42]. Moreover, Pcs can be easily grown as well-ordered films on various substrates with good compatibility in ultra high vacuum (UHV) systems [43–45]. Similar to copper(II) phthalocyanine (CuPc), copper(II) hexadecafluorophthalocyanine (F_16_CuPc), has the same central copper ion and the conjugated nitrogen atoms around it, but adds 16 strong electronegative fluorine groups, which result in different electronic structures (Fig. 1). Therefore, the two can represent a suitable model system to provide insight on the interaction mechanisms for conjugated organic materials utilized as sodium hosts or electrolyte additives in SMBs, especially for the fluorination promoted sodiophilic sites. Based on this, taking CuPc and F_16_CuPc as simplified model materials, we studied their interaction mechanisms with sodium metal by in-situ X-ray photoelectron spectroscopy (XPS), ultraviolet photoelectron spectroscopy (UPS), and density functional theory (DFT) calculations. We discovered that Na atoms prefer to interact with inner pyrrolic nitrogen atoms in CuPc, but with outer aza bridge nitrogen and symmetric fluorine atoms in F_16_CuPc. Moreover, with stronger electron affinity caused by the electron-withdrawing effect of fluorine atoms, the inner pyrrolic nitrogen atoms exhibit stronger interaction with sodium atoms at Na/F_16_CuPc interface as compared to Na/CuPc interface. In addition, the reduction of central copper ions in both CuPc and F_16_CuPc molecules were observed due to charge transfer from sodium. Our study presents a molecular-level understanding of the interaction process between Na and organic materials, aiming to guide the rational design of host materials and protective layers, by modifying the sodiophilic functional groups in organic materials.

## Experimental section

In-situ XPS and UPS experiments were conducted in a customer-designed UHV system composed of preparation and analysis chambers [46, 47], aiming to study the Na interaction process at Na/CuPc and Na/F_16_CuPc interfaces respectively. Two parts were included in each experiment to simulate the interaction process of hosts and the formation process of protective layers, including: (i) metallic sodium stepwise deposited on the organic films (10 nm) predeposited on the silicon substrates, simulating its interaction with hosts, and (ii) organic films deposited stepwise on metallic sodium (10 nm) film predeposited on a tungsten substrate, simulating the formation of protection layers. After each deposition of sodium or organic molecules in the preparation chamber (base pressure lower than 2 × 10^−8^ mbar^1^), the film was transferred directly to the analysis chamber (base pressure lower than 4 × 10^−10^ mbar) for XPS and UPS study. The thickness and surface morphology of the organic films predeposited on the silicon substrates were characterized through atomic force microscopy (AFM) using BRUKER Dimension Fast Scan AFM system. Moreover, the relevant DFT calculations were also conducted to further verify our conclusions.

Silicon and tungsten wafers were chosen as substrates for organic molecules and sodium metal films preparation respectively. Both were thoroughly degassed at around 400 °C in the UHV preparation chamber before organic molecules or sodium metal deposition. Vacuum sublimation purified CuPc, and F_16_CuPc molecules (> 99%, Luminescence Technology Corp), were thermally evaporated from separated Knudsen cells with temperatures of 290 °C and 300 °C respectively. Sodium metal was deposited from a SAES getter source with a 4.0 A direct current. The deposition thickness was obtained from XPS core-level intensities and measured through inelastic mean free path (IMFP) calculations [48]. All the organic molecules and sodium metal preparation and deposition processes were conducted in the same UHV preparation chamber.

XPS and UPS measurements were performed at room temperature in the analysis chamber via an X-ray source (Omicron DAR400) with Al kα (1486.7 eV) and Mg kα (1253.6 eV) dual anodes, an excitation source (Omicron VUV HIS 13) with He 1α (21.2 eV), and an electron analyzer (Omicron EA125) with resolution of 0.05 eV. A charge of − 5.0 V bias voltage was applied to test the secondary electron cut-off (SECO) of samples. For core-level spectra decomposition, CasaXPS software was used with a Shirley background, and a line shape of GL(50) (50% Gaussian plus 50% Lorentzian function).

For DFT studies, Gaussian 16a software was used with a B3LYP-D3BJ/6-311G(d,p) level of theory [49]. The adsorption energy of the optimized Na*-*CuPc (or Na-F_16_CuPc) complex was calculated by the energy difference between the complex and the sum of a free Na atom and a pristine organic molecule. The charge distribution was obtained via basin analysis using Multiwfn software package [50].

For actual performance comparison, the galvanostatic profiles of nucleation overpotential, and mass-transport controlled overpotential, were collected in the asymmetric cells with a current density of 0.5 mA/cm^2^ and an areal capacity of 1 mAh/cm^2^. The asymmetric cells contain a sodium metal as the counter electrode and Cu or CuPc–Cu or F_16_CuPc–Cu as the working electrode with an electrolyte containing 1 mol/L NaPF_6_ in Diglyme (DEGDME). The asymmetric cells were performed in CR2032 coin cells at room temperature with a single layer of commercial polypropylene (PP) separator.

## Results and discussion

A brief introduction to the characterized signals of pristine CuPc and F_16_CuPc is shown in Fig. 1. Owing to simultaneously π → π* transitions, satellite features (shake-up peaks) due to the energy loss of the photoelectrons are observed for both two molecules, which is consistent with previous reports [43, 51–59]. For CuPc [45, 52, 56–59], its C 1s peak contains two main components with different chemical environments. One is for pyrrolic carbon atoms at 285.9 eV (named as C_N_), and the other is for aromatic carbon atoms at 284.5 eV (named as C_C_). The energy shift of 1.4 eV between them is due to valence charge transfer from pyrrolic carbon to the more electronegative nitrogen atoms [43, 45]. Furthermore, the relative intensity of C_C_ and C_N_ components nearly equals to the theoretical value of 3:1, taking into account the satellites. Its N 1s region only contains one peak at 398.9 eV (named as N_C_), since the inner pyrrolic and outer aza bridge nitrogen atoms have a similar electronic environment and present similar binding energy in XPS [43, 53, 54, 57, 58]. Its Cu 2p region contains one component with a 2p_3/2_ signal at 935.6 eV (named as Cu(II)) originating from the central Cu(II) ions. For F_16_CuPc [54, 60], it has similar spectra for the N 1s and Cu 2p regions. N_C_ component is located at 399.1 eV, and Cu(II) component is located at 935.7 eV for 2p_3/2_ signal. Compared to CuPc, its C 1s region contains one more component at 287.3 eV (named as C_F_) originating from carbon atoms combined with fluorine atoms. The C_N_ and C_C_ components are located at 286.4 and 285.2 eV respectively. Moreover, the relative intensity of C_F_, C_N,_ and C_C_ components, nearly equals to the theoretical value of 2:1:1, taking into account the satellites. Its F 1s region contains only one component at 687.6 eV (named as F_C_), which originates from 16 fluorine atoms [54]. Notably, all same components in F_16_CuPc show higher binding energy than those in CuPc owing to the strong electron-withdrawing effect of fluorine atoms. In this case, other atoms, including carbon, nitrogen, and copper atoms, are more electropositive, thus have higher binding energy in the spectra.

### Na/CuPc

**Fig. 2:**
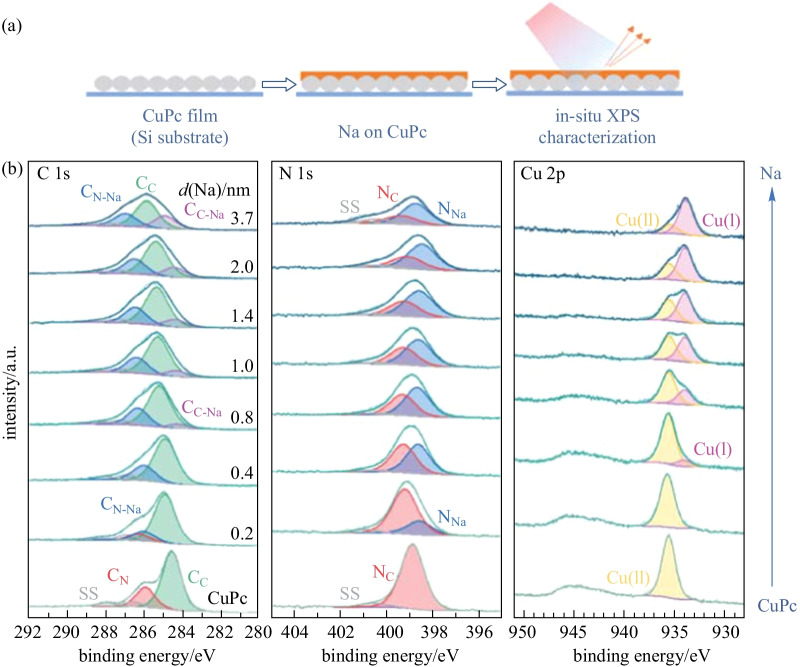
**a** Schematic of the deposition sequence. **b** XPS core-level spectra of CuPc with increasing Na deposition using a silicon foil as the substrate

The Na/CuPc interface was studied by in-situ XPS with the interaction process (i) of sodium deposited on CuPc, and the relevant spectra series are shown in Fig. 2. With 0.2 nm Na deposited, an overall N 1s asymmetric peak broadening is observed, and it is found that a new N_Na_ component appears at 0.6 eV lower binding energy (relative to N_C_) after peak decomposition. The relevant C_N-Na_ component is located at 0.3 eV lower binding energy (relative to C_N_) signal in C 1s region, while C_C_ signal remains unchanged. It indicates that sodium first interacts with nitrogen atoms and transfers electrons to the connected pyrrole carbon atoms. Next with 0.4 nm Na deposited, a new Cu(I) component appears at 1.60 eV lower binding energy (relative to Cu(II)) in the Cu 2p_3/2_ region, indicating the reduction of Cu(II) to Cu(I) ions owing to the charge transfer from sodium [52]. It should be noted that we cannot identify whether the reduced component is Cu(I) or Cu(0) from 2p_3/2_ signal only, since they are separated by the same binding energy difference with Cu(II) [53, 58]. In this way, we also take the Cu LMM auger spectrum (Additional file 1: Fig. S5) into account; and it can be concluded that the reduced product is Cu(I) ion by calculating the auger parameter [61]. Moreover, half nitrogen atoms interact with sodium to form N_Na_, and nearly all connected carbon atoms receive electrons to form C_N-Na_. According to DFT calculations (to be discussed in detail at the end of the paper), we suggest that sodium first interacts with the inner pyrrolic nitrogen atoms and transfers electrons to reduce Cu(II) ions simultaneously. Following that, more N_Na_ component appears to dominate in the N 1s region with thicker sodium deposited, indicating that sodium also interacts with the outer aza bridge nitrogen atoms. A new component C_C-Na_ is also observed at 0.9 eV lower binding energy (relative to C_C_). Through DFT calculations (to be discussed in detail at the end of the paper), we suggest that when the sodium atom interacts with the outer aza bridge nitrogen atom, its position is close to the benzene ring on one side and transfers electrons to aromatic carbon atoms, resulting in the formation of C_C-Na_ signal. It is also observed in the previous report about K/MnPc interface [62]. In this way, for the Na/CuPc interface, it can be concluded that sodium atoms interact with the inner pyrrolic nitrogen atoms of CuPc first, and then with the outer aza bridge nitrogen atoms. Furthermore, benzene rings receive electrons from sodium, owing to the sodium anchoring position. Moreover, Cu(II) ions are reduced to Cu(I) ions during the process.

**Fig. 3:**
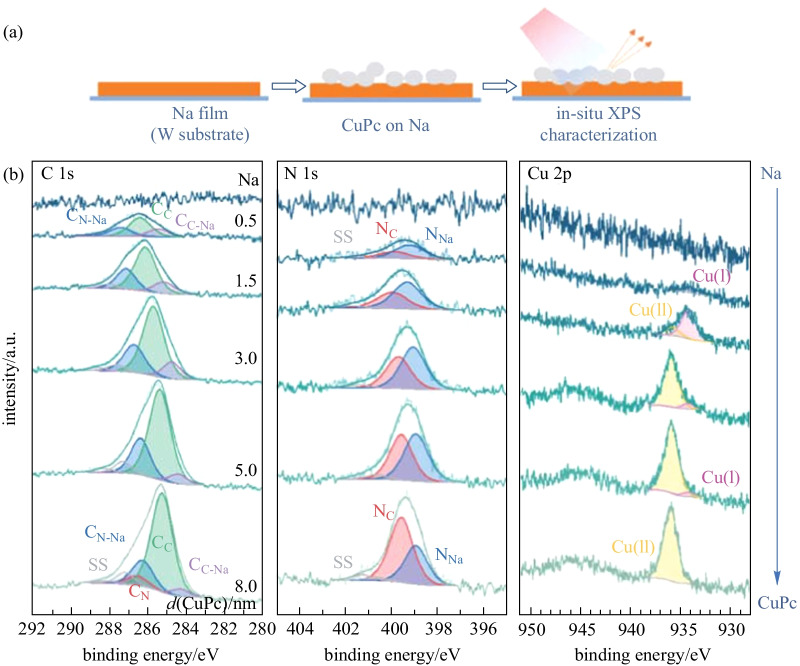
**a** Schematic of the deposition sequence. **b** XPS core-level spectra of Na with increasing CuPc deposition using a tungsten foil as the substrate

The interaction process (ii) of CuPc deposited on metallic sodium was also investigated (Fig. 3). Similar conclusions can be reached, demonstrating that interfacial interaction is identical and independent of the deposition sequence. With 0.5 nm CuPc deposited, over half nitrogen atoms interact with sodium to form N_Na_ in the N 1s region, and C 1s peak consists of three components originating from C_N-Na_, C_C,_ and C_C-Na_ of Na interacted CuPc respectively. It indicates that all carbon atoms connected with nitrogen atoms receive electrons when the inner pyrrolic and outer aza bridge nitrogen atoms interact with sodium, and part of carbon atoms also receive electrons indirectly, owing to the sodium interaction position. Besides, all Cu 2p_3/2_ signals originate from Cu(I) ions owing to the charge transfer from sodium. With more CuPc deposited, the ratio of C_C_ to C_C-Na_, N_C_ to N_Na,_ and Cu(II) to Cu(I) component increases gradually. And with 8.0 nm CuPc deposited, the original C_N_ signal of CuPc is detected, indicating that the Na-CuPc interaction only takes place near the interface region and the reacted CuPc molecules are gradually covered by the original CuPc molecules. Consequently, both Na on CuPc and CuPc on Na interactions have same modes as the interaction process takes place only at the interface.

The evolution of electronic structures at Na/CuPc interface was also measured by in-situ UPS characterizations. As shown in Additional file 1: Fig. S2, the valance band (VB) shape of CuPc is in great accordance with previous reports [54, 55, 57, 58]. With increasing Na deposition, the work function measured from SECO gradually decreases due to the formation of reacted CuPc with electron receiving from Na [56, 57, 63]. Meanwhile, the original CuPc peak broadens and weakens in the VB region. Moreover, the top of the VB spectrum, which originates from the highest occupied molecular orbital (HOMO) of CuPc, is located at 1.47 eV below the Fermi level (*E*_F_) [43, 52, 57]. With sodium deposition, a new lowest unoccupied molecular orbital (LUMO)-derived signal appears at 0.74 eV. This state shows clear evidence of the charge transfer from sodium to the LUMO of CuPc, leading to the formation of occupied electronic levels in the energy gap [55, 57–59, 63–65].

In addition, UPS spectra for Na grown on W, with increasing CuPc deposition (Additional file 1: Fig. S3), show similar results in a reverse process. After the deposition of CuPc, the work function measured from SECO gradually increases, then it remains nearly unchanged until the molecular layer thickness is higher than 5.0 nm since the surface molecules are nearly all unreacted CuPc. In the VB region, the original Na peak broadens and weakens, and great shape change takes place with CuPc deposition, which transfers gradually to be similar to that of CuPc. In the HOMO edge region, a new HOMO signal appears and then gradually shifts to the lower binding energy side (at 0.82 eV with 8.0 nm CuPc deposited), due to the charge transfer from Na to CuPc LUMO. And another peak appears at 2.00 eV, which is proposed to originate from the HOMO state of pristine CuPc, considering its binding energy difference with other main VB peaks.

### Na/F_16_CuPc

**Fig. 4:**
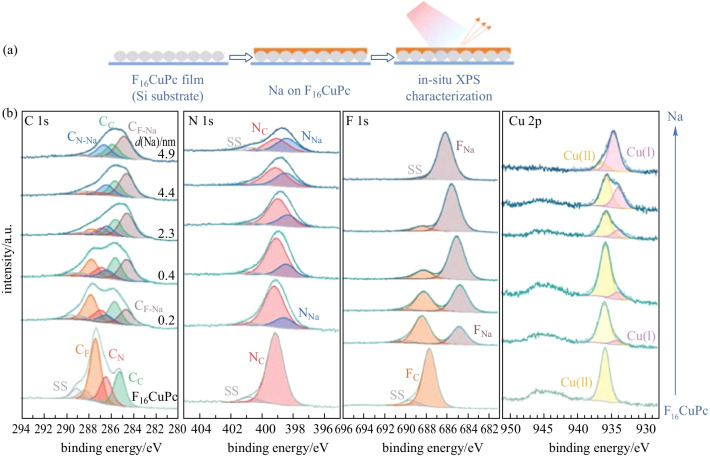
**a** Schematic of the deposition sequence. **b** XPS core-level spectra of F_16_CuPc with increasing Na deposition using a silicon foil as the substrate

To study the fluorination effected sodiophilic sites, the Na/F_16_CuPc interface was investigated. The interaction process (i) of Na deposited on F_16_CuPc films was first studied (Fig. 4). With 0.2 nm Na deposited, interaction between Na and nitrogen atoms is observed with the formation of N_Na_ component at 0.6 eV lower binding energy (relative to N_C_), and the relevant C_N-Na_ component at 0.5 eV lower binding energy (relative to C_N_). The signal of Cu(I) ions appears owing to the reduction of Cu(II) ions. Besides, a new F_Na_ component at 3.6 eV lower binding energy (relative to F_C_), and the relevant C_F-Na_ signal at 3.1 eV lower binding energy (relative to C_F_), appear in F 1s and C 1s region respectively, indicating the ionic interaction between Na and fluorine atoms. According to DFT calculations (to be discussed in detail at the end of the paper), we suggest that in this step, sodium prefers to interact with the outer aza bridge nitrogen atoms, due to the neighboring strong electronegative fluorine atoms. We suppose that the deposited sodium atoms transfer electrons to both nitrogen and fluorine atoms at the same time. With increasing Na thickness, more nitrogen and fluorine atoms take part in the interaction and more Cu(II) ions are reduced. Notably, with 4.9 nm Na deposited, only half nitrogen atoms are shown as N_Na_ component, but nearly all fluorine atoms are shown as F_Na_ component, indicating that sodium prefers to interact with fluorine atoms and only interacts with outer aza bridge nitrogen atoms in F_16_CuPc. It should also be mentioned that no C_C-Na_ signal is observed during the Na deposition process, which is different from that at Na/CuPc interface. Through DFT calculations (to be discussed in detail at the end of the paper), we suggest that it is related to the Na interaction position: when sodium atom interacts with the outer aza bridge nitrogen atom, Na locates at the bridge site between two neighboring benzene rings without obvious charge transfer to the aromatic carbon atoms. Consequently, at Na/F_16_CuPc interface, the interaction preferentially takes place between sodium and fluorine atoms as well as the outer aza bridge nitrogen atoms. Similar to Na/CuPc interface, central Cu(II) ions are reduced to Cu(I) ions.

**Fig. 5:**
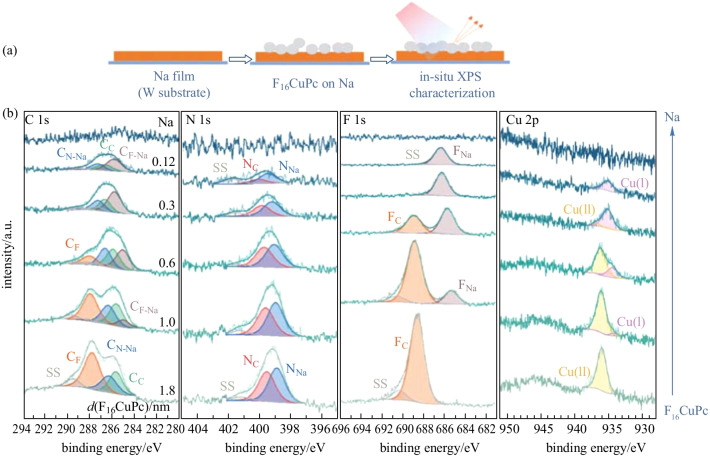
**a** Schematic of the deposition sequence. **b** XPS core-level spectra of Na with increasing F_16_CuPc deposition using a tungsten foil as the substrate

**Fig. 6:**
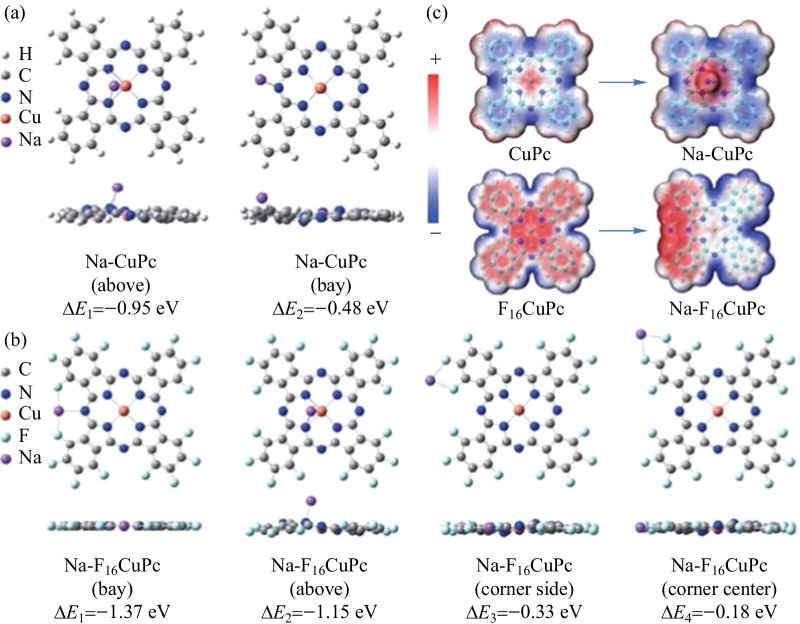
Adsorption energy (Δ*E*) of optimized **a** Na-CuPc and **b** Na-F_16_CuPc structures. **c** Charge distribution of F_16_CuPc, Na-F_16_CuPc, CuPc, and Na-CuPc

The interaction process (ii) of F_16_CuPc deposited on metallic sodium was also studied (Fig. 5). Similarly, the reversed deposition sequence has little influence on the interfacial interaction, except that the interaction between sodium and inner pyrrolic nitrogen atoms is detected in this case. Specifically, with 0.12 nm F_16_CuPc deposited on metallic Na film, the C 1s peak consists of three components: C_F-Na_, C_N-Na,_ and C_C_, which means that all carbon atoms linked with nitrogen or fluorine atoms receive electrons from sodium atoms. In contrast, the other carbon atoms remain intact with that of pristine F_16_CuPc. Over half the nitrogen atoms are shown as N_Na_ component and all fluorine atoms are shown as F_Na_ component, corresponding well to the C 1s signal. It indicates that both kinds of nitrogen atoms and all fluorine atoms take part in the interaction process owing to the presence of abundant sodium atoms. Also, the Cu 2p_3/2_ signal originates from Cu(I) ions due to the reduction caused by the charge transfer. Then with 0.6 nm F_16_CuPc deposited, the original C_F_ signal of F_16_CuPc appears, indicating that the interaction only takes place in the near interface region. The reacted F_16_CuPc molecules are gradually covered under the unreacted F_16_CuPc [60]. With further F_16_CuPc deposited, nearly all C_F-Na_ and F_Na_ transform to C_F_ and F_C_ respectively, while the signals of C_N-Na_ and C_C_ remain unchanged. Another observation is that there is always over half nitrogen atoms shown as N_Na_. According to the charge distribution calculation (Fig. 6), we suggest that it is related to the strong electron-withdrawing effect of fluorine atoms. In other words, since the inner pyrrolic nitrogen atoms are more electropositive with stronger electron affinity and sodium atoms are abundant around the molecules, the electron transfer from sodium to inner pyrrolic nitrogen atoms is strengthened. Consequently, when F_16_CuPc molecules are deposited on Na, the interaction between sodium and inner pyrrolic nitrogen atoms is also observed, which is strengthened due to the strong electron-withdrawing effect of fluorine atoms.

The evolution of electronic structures at Na/F_16_CuPc interface was also measured by in-situ UPS characterizations. As shown in Additional file 1: Fig. S4, the VB shape of F_16_CuPc and the position of HOMO (spectral weight maximum located at 1.44 eV below the *E*_F_), are consistent with the prior reports [54, 66]. With increasing Na deposition, the gradual decrease of work function measured from SECO is observed due to the formation of reacted F_16_CuPc with electron receiving from Na. Meanwhile, the original F_16_CuPc peak broadens and weakens in the VB region. A new LUMO-derived signal is observed at 0.68 eV, which is related to the charge transfer from sodium to the LUMO of F_16_CuPc [60].

In addition, UPS spectra for Na grown on W with increasing F_16_CuPc deposition (Additional file 1: Fig. S5) show similar results in a reverse process. After the deposition of F_16_CuPc, the work function measured from SECO gradually increases to 4.32 eV with 1.8 nm F_16_CuPc deposited, becoming gradually close to that of unreacted F_16_CuPc (4.81 eV). In the VB region, the original Na peak broadens and weakens, and great shape change takes place with F_16_CuPc deposition, which transforms gradually to be the similar shape of F_16_CuPc. Notably, in the HOMO edge region, with increasing F_16_CuPc deposition, two new LUMO-derived signals appear at the lower binding energy side (0.86 and 1.58 eV with 1.8 nm F_16_CuPc deposited), which is more than that in the interaction process of Na deposited on F_16_CuPc. It may relate to the relative abundant sodium atoms, which are able to provide enough electrons to fill two unoccupied orbitals of molecules. Besides, another peak appears at 2.25 eV, which is proposed to originate from the HOMO state of pristine F_16_CuPc, considering its binding energy difference with other main signals in the VB spectrum.

### DFT calculation

DFT calculations for Na adsorption on a single Pc molecule were carried out to further study the interaction order of different sites. Two possible optimized structures of Na-CuPc complex and four possible optimized structures of Na-F_16_CuPc complex were studied as shown in Fig. 6a, b respectively. For Na-CuPc, the above position of complex displays the larger adsorption energy (Δ*E* =  − 0.95 eV), indicating that sodium prefers to interact with the inner pyrrolic nitrogen atoms and then with the outer aza bridge nitrogen atoms. The optimized bay position of Na-CuPc complex also shows that Na is adsorbed close to one side of benzene ring when it interacts with the outer aza bridge nitrogen atoms, thus it is able to transfer charge to the benzene rings resulting in the formation of C_C-Na_ in the C 1s spectrum. For Na-F_16_CuPc, the bay position of complex displays the largest adsorption energy (Δ*E* =  − 1.37 eV). It indicates that Na is first anchored by the outer aza bridge nitrogen atom and two symmetric fluorine atoms, and then interacts with the inner pyrrolic nitrogen atoms and the last fluorine atoms. And the optimized bay position of Na-F_16_CuPc complex, also shows that Na is anchored at the bridge site between two neighboring benzene rings and no obvious charge is transferred to the aromatic carbon atoms, thus no C_C-Na_ signal is observed at the Na/F_16_CuPc interface. Moreover, from the charge distribution of pristine F_16_CuPc, it is observed that the inner pyrrolic nitrogen atom sites are highly positive charged, confirming that the interaction between Na and the inner pyrrolic nitrogen atoms is strengthened at the Na/F_16_CuPc interface.

## Conclusion

Fluorination promoted sodiophilic sites were investigated by in-situ XPS/UPS and DFT calculations through the comparison of the model systems of CuPc and F_16_CuPc. The proposed Na interaction behaviors can be described as follows: Na atoms prefer to interact with the inner pyrrolic N atoms in CuPc, whereas they prefer to interact with the outer aza bridge N atoms with the assistance of two neighboring symmetric F atoms in F_16_CuPc. Moreover, due to the stronger electron affinity of inner pyrrolic nitrogen atoms of F_16_CuPc caused by the electron-withdrawing effect of fluorine atoms, stronger interaction between sodium atoms and inner pyrrolic nitrogen atoms is observed at Na/F_16_CuPc interface. In addition, the reduction of central Cu(II) to Cu(I) ions in both F_16_CuPc and CuPc molecules is observed. Our model studies unravel the Na interaction process at Na/CuPc and Na/F_16_CuPc interfaces, especially the effect of fluorination on sodiophilic sites, which provide insights into the radical design of fluorine-containing electrolyte additives and hosts for the protection of sodium metal anode.

## Supplementary Information

**Additional file 1.** Thickness and surface morphology of CuPc and F_16_CuPc films characterized by AFM; UPS spectra for Na deposited on CuPc (or F_16_CuPc) using silicon wafers as substrates and CuPc (or F_16_CuPc) deposited on Na using tungsten wafers as substrates; XPS spectra of Cu LMM auger and Cu 2p regions for pristine CuPc and after 3.7 nm Na deposition; voltage profiles of galvanostatic electrodeposition of Na for Na|Cu, Na|CuPc-Cu, and Na|F_16_CuPc-Cu asymmetric cells and the summary of the mass-transport controlled overpotential (*η*_1_) and the nucleation overpotential (*η*_2_) for these cells; detailed XPS peak fitting parameters for CuPc (or F_16_CuPc) with increasing Na deposition and Na with increasing CuPc (or F_16_CuPc) deposition.

## Footnotes

1 1 mbar = 100 Pa.
