# Supplementary material for: Probing fluorination promoted sodiophilic sites with model systems of F16CuPc and CuPc
Source: Front Optoelectron. 2022 Apr 28;15(1):19. doi: 10.1007/s12200-022-00026-3 (PMC9756233; doi:10.1007/s12200-022-00026-3)
Supplement: Supplementary file 1 — Additional file 1. Thickness and surface morphology of CuPc and F16CuPc films characterized by AFM; UPS spectra for Na deposited on CuPc (or F16CuPc) using silicon wafers as substrates and CuPc (or F16CuPc) deposited on Na using tungsten wafers as substrates; XPS spectra of Cu LMM auger and Cu 2p regions for pristine CuPc and after 3.7 nm Na deposition; voltage profiles of galvanostatic electrodeposition of Na for Na|Cu, Na|CuPc-Cu, and Na|F16CuPc-Cu asymmetric cells and the summary of the mass-transport controlled overpotential (η1) and the nucleation overpotential (η2) for these cells; detailed XPS peak fitting parameters for CuPc (or F16CuPc) with increasing Na deposition and Na with increasing CuPc (or F16CuPc) deposition. [file 12200_2022_26_MOESM1_ESM.pdf]

Supporting Information

Additional file 1 for

Probing fluorination promoted sodiophilic sites with model systems of $F_{16}CuPc$ and CuPc

Yuan Liu,[a, b 1] Xu Lian,[b, c 1] Zhangdi Xie,[b] Jinlin Yang,[b] Yishui Ding,[a, b] Wei Chen[a, b, d *]

[a] Joint School of National University of Singapore and Tianjin University, International Campus of Tianjin University, Binhai New City, Fuzhou 350207, PR China

[b] Department of Chemistry, National University of Singapore, 3 Science Drive 3, Singapore 117543, Singapore

[c] Centre for Advanced 2D Materials, National University of Singapore, 6 Science Drive 2, Singapore 117546, Singapore

[d] Department of Physics, National University of Singapore, 2 Science Drive 3, 117542, Singapore

*Corresponding Author E-mail: phycw@nus.edu.sg (Wei Chen).

[1]These authors contributed equally to this paper.

This Additional file includes:

Thickness and surface morphology of CuPc and $F_{16}CuPc$ films characterized by AFM; *in-situ* UPS spectra for sequential Na deposition on CuPc (or $F_{16}CuPc$) using silicon wafers as substrates and sequential CuPc (or $F_{16}CuPc$) deposition on Na using tungsten wafers as substrates; XPS spectra of Cu LMM auger and Cu 2p regions for pristine CuPc and after 3.7 nm Na deposition; voltage profiles of galvanostatic electrodeposition of Na for Na|Cu, Na|CuPc-Cu, and Na|$F_{16}CuPc$-Cu asymmetric cells and the summary of the mass-transport controlled overpotential (η_1) and the nucleation overpotential (η_2) for these cells; detailed XPS peak fitting parameters for CuPc (or $F_{16}CuPc$) with increasing Na deposition and Na with increasing CuPc (or $F_{16}CuPc$) deposition.

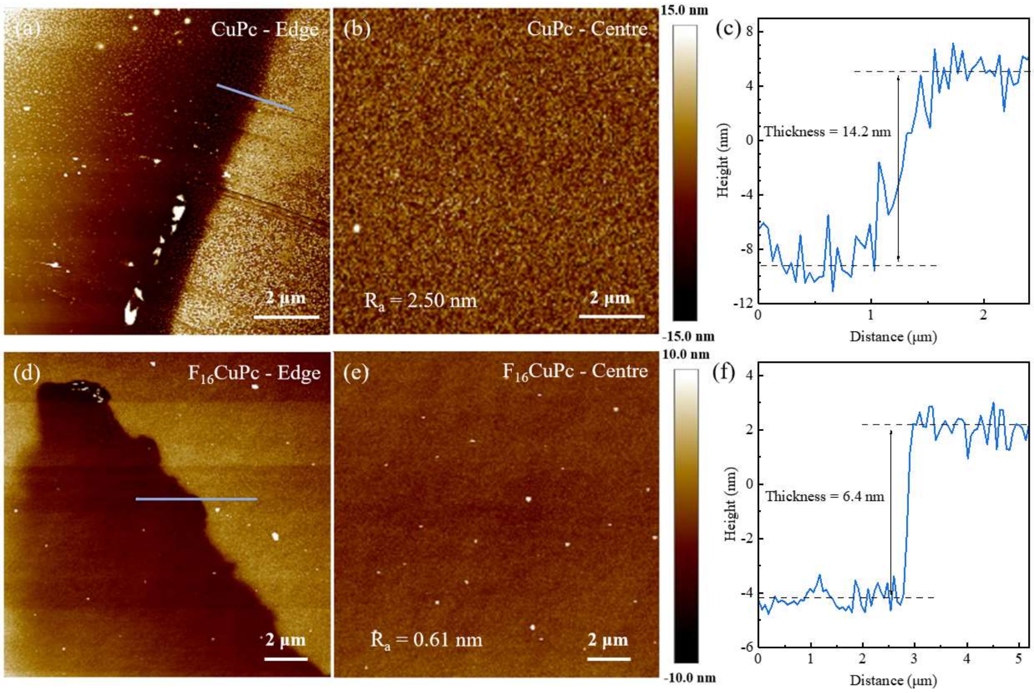

Fig. S1 Thickness and surface morphology of **a–c** CuPc and **d–f** F_{16}CuPc films characterized by AFM.

We characterized the thickness and surface morphology of CuPc and F_{16}CuPc films on Si substrates via atomic force microscopy (AFM). Thicknesses of CuPc and F_{16}CuPc films were about 14.2 and 6.4 nm respectively. The average roughness (R_a) of both CuPc and F_{16}CuPc films was lower than 3.00 nm, indicating a smooth surface for both samples.

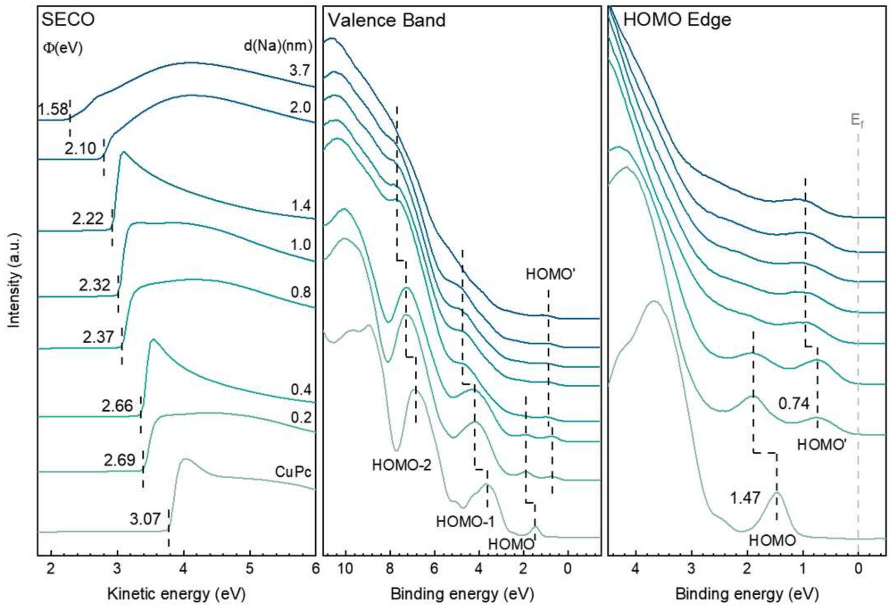

Fig. S2 UPS spectra of SECO region, VB region and HOMO edge region for CuPc grown on a silicon foil with increasing Na thickness.

In-situ UPS was conducted to study the electronic evolution at Na/CuPc interface. The valance band (VB) shape and the position of highest occupied molecular orbital (HOMO) (spectral weight maximum located at 1.47 eV below the Fermi level) are in good agreement with previous reports[1]. With increasing Na deposition, the work function measured from SECO gradually decreases due to the formation of reacted CuPc with electron receiving from Na[1-3]. Meanwhile, the original CuPc peak broadens and weakens in the VB region. Besides, a new LUMO-derived signal appears near E_F at 0.74 eV, showing clear evidence of the charge transfer from sodium to the LUMO of CuPc[1, 3].

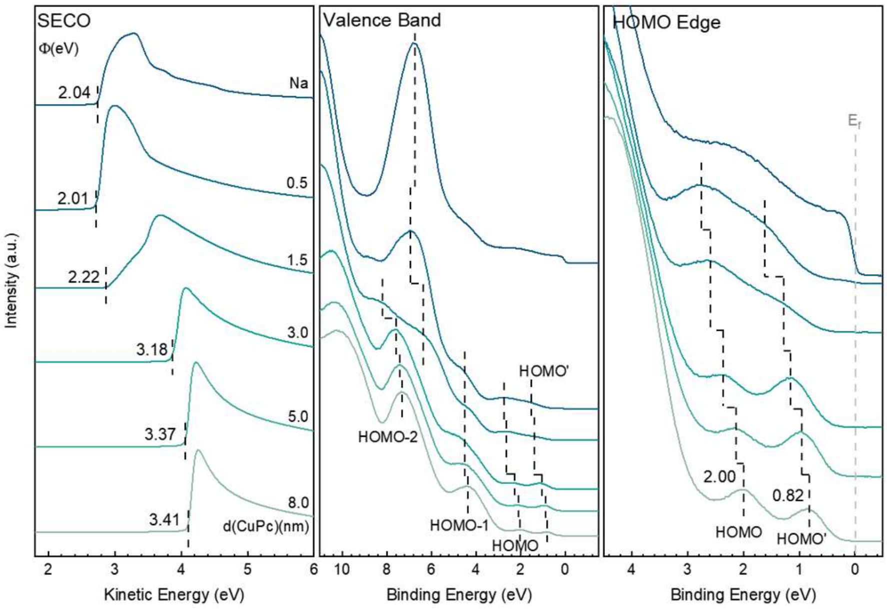

Fig. S3 UPS spectra of SECO region, VB region and HOMO edge region for Na grown on a tungsten foil with increasing CuPc thickness.

UPS spectra for Na grown on W with increasing CuPc deposition show similar results in a reverse process. After the deposition of CuPc, the work function measured from SECO gradually increases and then remains nearly unchanged with nearly all unreacted CuPc on the surface. In the VB region, the original Na peak broadens and weakens and transfers to be similar to that of CuPc gradually. In the HOMO edge region, a new HOMO signal appears and then gradually shifts to the lower binding energy side (at 0.82 eV with 8.0 nm CuPc deposited) due to charge transfer from Na to CuPc LUMO. And another peak appears at 2.00 eV, which is thought to originate from the HOMO state of pristine CuPc considering its binding energy difference with other main VB peaks.

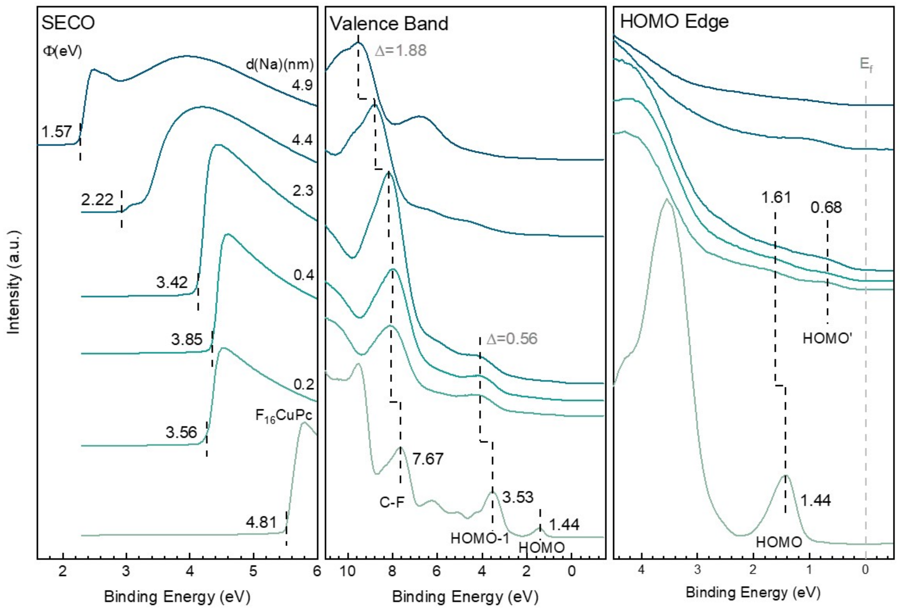

Fig. S4 UPS spectra of SECO region, VB region and HOMO edge region for $F_{16}CuPc$ grown on a silicon foil with increasing Na thickness.

The evolution of electronic structures at Na/$F_{16}CuPc$ interface was also measured by *in-situ* UPS characterizations. The VB shape of $F_{16}CuPc$ and the position of HOMO (spectral weight maximum located at 1.44 eV below the E_F) are in good agreement with the previous reports[4,5]. With increasing Na deposition, the work function measured from SECO gradually decreases due to the formation of reacted $F_{16}CuPc$ with electron receiving from Na. Meanwhile, the original $F_{16}CuPc$ peak broadens and weakens in the VB region. A new LUMO-derived signal appears near the Fermi level at 0.68 eV due to the charge transfer from sodium to the LUMO of $F_{16}CuPc$[6].

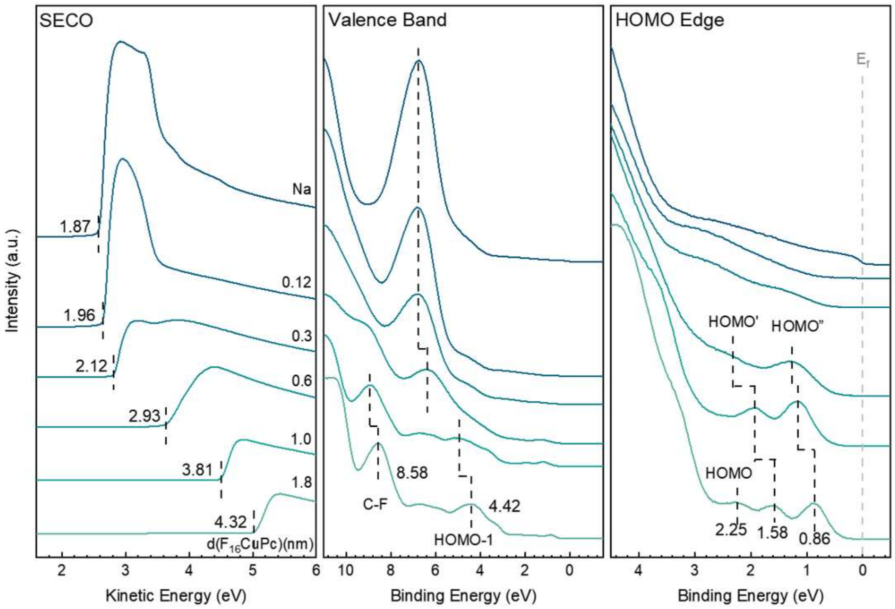

Fig. S5 UPS spectra of SECO region, VB region and HOMO edge region for Na grown on a tungsten foil with increasing $F_{16}CuPc$ thickness.

UPS spectra for Na grown on W with increasing $F_{16}CuPc$ deposition show similar results in a reverse process. After the deposition of $F_{16}CuPc$, the work function measured from SECO gradually increases and becomes gradually close to that of unreacted $F_{16}CuPc$ (4.81 eV). In the VB region, the original Na peak broadens and weakens and transforms to be the similar shape of $F_{16}CuPc$ gradually. In the HOMO edge region, with increasing $F_{16}CuPc$ deposition, two new LUMO-derived signals appear at the lower binding energy side (0.86 and 1.58 eV with 1.8 nm $F_{16}CuPc$ deposited). It may relate to the relative abundant sodium atoms, which are able to provide enough electrons to fill two unoccupied orbitals of molecules. Besides, another peak appears at 2.25 eV, which is proposed to originate from the HOMO state of pristine $F_{16}CuPc$ considering its binding energy difference with other main signals in the VB spectrum.

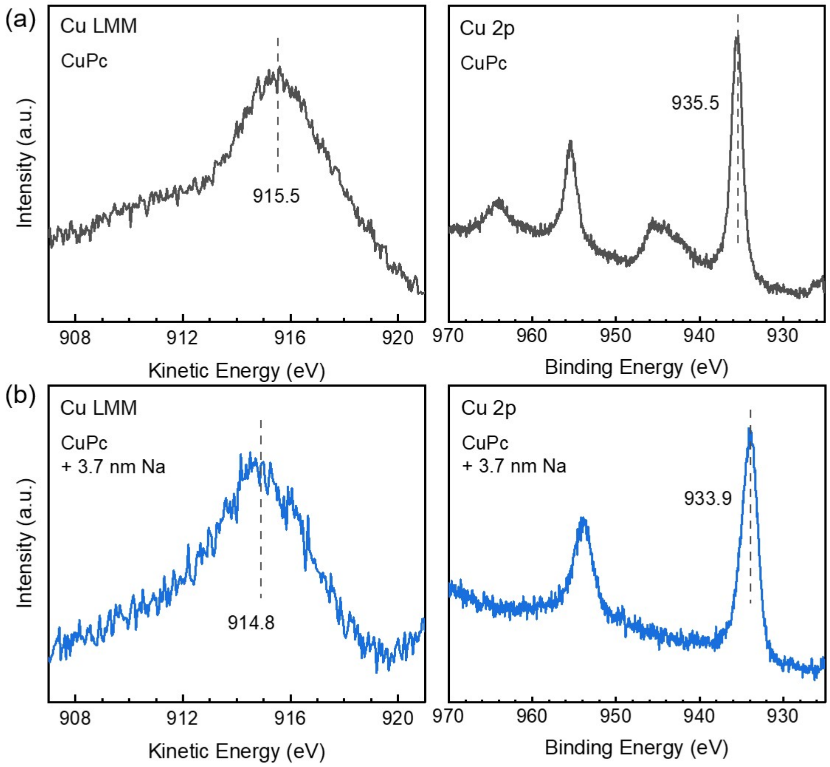

Fig. S6 XPS spectra of Cu LMM auger and Cu 2p core-level regions for (a) pristine CuPc and (b) after 3.7 nm Na deposition.

Cu LMM auger and 2p spectra are both taken into account to study the interaction process of central copper ions. For pristine CuPc, the Cu $2p_{3/2}$ signal of Cu(II) ions is located at 935.5 eV binding energy and the relevant LMM signal is mainly located at 915.5 eV kinetic energy. According to previous reports, its auger parameter (kinetic energy of LMM plus binding energy of $2p_{3/2}$) equals 1851.0 eV which is consistent with that of CuO[7]. After 3.7 nm Na deposition, the $2p_{3/2}$ signal becomes located at 933.9 eV binding energy and the relevant LMM signal is mainly located at 914.8 eV kinetic energy. Accordingly, its auger parameter equals 1848.7 eV which is close to that of Cu_2O[7]. Moreover, the Cu LMM auger peak of metallic Cu(0) is reported at higher kinetic energy of 918.4 eV[8]. Thus the reduced product of Cu(II) ions can be identified as Cu(I) ions[9].

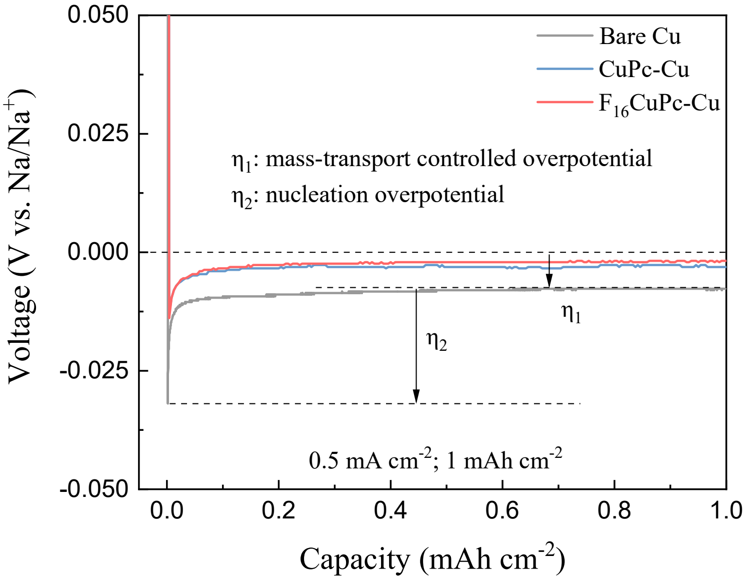

Fig. S7 Voltage profiles of galvanostatic electrodeposition of Na for Na|Cu, Na|CuPc-Cu, and Na|F_{16}CuPc-Cu asymmetric cells measured at a current density of 0.5 mA/cm^2.

Na|Cu, Na|CuPc-Cu, and Na|F_{16}CuPc-Cu asymmetric cells were fabricated and tested with a current density of 0.5 mA/cm^2 and an areal capacity of 1 mAh/cm^2. When Na is deposited on the Cu foil, there mainly exist two steps: nucleation process and growth process. Due to the presence of concentration polariton of Na^+ during nucleation process and diffusion barrier of Na atom during growth process, nucleation overpotential (η_2) and mass-transport controlled overpotential (η_1) will be generated, respectively[10,11]. As shown in Fig. S7, with the coating of CuPc and F_{16}CuPc on the Cu foil, the nucleation overpotential and mass-transport controlled overpotential of Na|CuPc-Cu, and Na|F_{16}CuPc-Cu asymmetric cells are greatly reduced compared to that of Na|Cu asymmetric cell. The reduced overpotential can be contributed to the abundant nitrogen and fluorine sites, which can facilitate the formation of Na-N and Na-F and then enable homogenous sodium ion flux.

Table S1 Summary of the mass-transport controlled overpotential (η_1) and the nucleation overpotential (η_2) for the Na|Cu, Na|CuPc-Cu, and Na|F_{16}CuPc-Cu asymmetric cells.

	Bare Cu	CuPc-Cu	F_{16}CuPc-Cu
η_1/mV	7.7	3.1	1.8
η_2/mV	24.2	9.9	12.1

Table S2 Detailed peak fitting parameters in C 1s, N 1s and Cu 2p$_{3/2}$ XPS core-level spectra for CuPc with increasing Na deposition.

Na/CuPc		C 1s				N 1s		Cu 2p	
		**C_N**	**$C_{N\text{-}Na}$**	**C_C**	**$C_{C\text{-}Na}$**	**N_C**	**N_{Na}**	**Cu(II)**	**Cu(I)**
**3.7 nm**	BE/eV	/	287.0	285.9	284.9	399.4	398.8	935.5	933.9
	FWHM	/	1.40	1.40	1.30	1.60	1.40	1.70	1.70
	Area/%	/	25.0	51.2	23.8	31.3	68.7	20.3	79.7
**2.0 nm**	BE/eV	/	286.5	285.4	284.4	399.1	398.5	935.6	934.0
	FWHM	/	1.36	1.40	1.30	1.60	1.45	1.70	1.70
	Area/%	/	25.0	59.2	15.8	34.5	65.5	32.0	68.0
**1.4 nm**	BE/eV	/	286.4	285.3	284.4	399.3	398.6	935.6	934.0
	FWHM	/	1.32	1.40	1.30	1.50	1.45	1.70	1.70
	Area/%	/	25.0	63.2	11.8	37.0	63.0	42.0	58.0
**1.0 nm**	BE/eV	/	286.4	285.3	284.3	399.3	398.6	935.6	934.0
	FWHM	/	1.30	1.40	1.30	1.35	1.33	1.70	1.70
	Area/%	/	25.0	65.2	9.8	40.0	60.0	49.7	50.3
**0.8 nm**	BE/eV	/	286.3	285.2	284.3	399.3	398.7	935.6	934.0
	FWHM	/	1.22	1.40	1.20	1.25	1.25	1.70	1.70
	Area/%	/	25.0	68.8	6.2	41.8	58.2	67.5	32.5
**0.4 nm**	BE/eV	/	286.0	284.9	/	399.3	398.6	935.7	934.1
	FWHM	/	1.32	1.40	/	1.25	1.20	1.69	1.70
	Area/%	/	25.0	75.0	/	50.0	50.0	88.5	11.5

**0.2 nm**	BE/eV	286.3	286.0	284.9	/	399.2	398.6	935.7	/
	FWHM	1.20	1.30	1.29	/	1.30	1.26	1.70	/
	Area/%	10.1	14.9	75.0	/	77.0	23.0	100.0	/
**CuPc**	BE/eV	285.9	/	284.5	/	398.9	/	935.6	/
	FWHM	1.10	/	1.16	/	1.21	/	1.60	/
	Area/%	25.0	/	75.0	/	100.0	/	100.0	/

Table S3 Detailed peak fitting parameters in C 1s, N 1s and Cu $2p_{3/2}$ XPS core-level spectra for Na with increasing CuPc deposition.

**Na/CuPc**		**C 1s**				**N 1s**		**Cu 2p**	
		**C_N**	**$C_{N\text{-}Na}$**	**C_C**	**$C_{C\text{-}Na}$**	**N_C**	**N_{Na}**	**Cu(II)**	**Cu(I)**
**Na**	BE/eV	/	/	/	/	/	/	/	/
	FWHM	/	/	/	/	/	/	/	/
	Area/%	/	/	/	/	/	/	/	/
**0.5 nm**	BE/eV	/	287.4	286.4	285.5	399.9	399.2	/	934.0
	FWHM	/	1.30	1.30	1.23	1.60	1.53	/	1.60
	Area/%	/	25.0	54.1	20.9	34.5	65.5	/	100.0
**1.5 nm**	BE/eV	/	287.1	286.1	285.2	400.0	399.3	935.8	934.2
	FWHM	/	1.19	1.30	1.30	1.55	1.44	1.60	1.60
	Area/%	/	25.0	59.0	16.0	39.4	60.6	23.2	76.8
**3.0 nm**	BE/eV	/	286.7	285.7	284.8	399.7	399.1	936.0	934.3
	FWHM	/	1.30	1.30	1.10	1.27	1.27	1.60	1.39
	Area/%	/	25.0	62.2	12.8	42.9	57.1	91.5	8.5
**5.0 nm**	BE/eV	/	286.4	285.4	284.4	399.6	399.0	935.9	934.2
	FWHM	/	1.16	1.30	1.10	1.19	1.26	1.60	1.60
	Area/%	/	25.0	68.6	6.4	47.6	52.4	93.7	6.3
**8.0 nm**	BE/eV	286.5	286.3	285.3	284.3	399.6	399.0	936.0	/
	FWHM	1.30	1.27	1.30	1.10	1.21	1.16	1.60	/
	Area/%	6.8	18.2	70.4	4.6	63.5	36.5	100.0	/

Table S4 Detailed peak fitting parameters in C 1s, F 1s, N 1s and Cu $2p_{3/2}$ XPS core-level spectra for $F_{16}CuPc$ with increasing Na deposition.

**Na/F_{16}CuPc**		**C 1s**					**N 1s**		**F 1s**		**Cu 2p**	
		**C_F**	**C_N**	**$C_{N\text{-}Na}$**	**C_C**	**$C_{F\text{-}Na}$**	**N_C**	**N_{Na}**	**F_C**	**F_{Na}**	**Cu(II)**	**Cu(I)**
**4.9 nm**	BE/eV	287.9	/	286.6	285.8	284.8	399.0	398.4	688.6	686.1	936.3	934.5
	FWHM	1.30	/	1.56	1.42	1.60	1.60	1.50	1.60	1.66	1.80	1.80
	Area/%	1.7	/	25.0	25.0	48.3	50.0	50.0	3.3	96.7	17.6	82.4
**4.4 nm**	BE/eV	287.7	286.8	286.3	285.6	284.6	399.1	398.5	688.3	685.5	935.6	933.8
	FWHM	1.33	1.50	1.50	1.30	1.50	1.70	1.50	1.80	1.69	1.80	1.80
	Area/%	5.1	5.1	19.9	25.0	44.9	60.2	39.8	10.3	89.7	58.4	41.6
**2.3 nm**	BE/eV	287.7	286.8	286.3	285.6	284.6	399.0	398.3	688.2	685.0	935.6	933.8
	FWHM	1.30	1.30	1.30	1.30	1.50	1.50	1.38	1.80	1.71	1.80	1.80
	Area/%	8.9	10.9	14.1	25.0	41.1	71.7	28.3	17.7	82.3	78.6	21.4
**0.4 nm**	BE/eV	287.7	286.8	286.3	285.6	284.6	399.1	398.4	688.2	684.7	935.7	933.9
	FWHM	1.38	1.50	1.50	1.30	1.50	1.50	1.30	1.80	1.73	1.80	1.80
	Area/%	21.4	13.8	11.2	25.0	28.6	77.7	22.3	42.8	57.2	87.9	12.1
**0.2 nm**	BE/eV	287.8	286.8	286.4	285.6	284.6	399.2	398.5	688.3	684.8	935.8	934.0
	FWHM	1.39	1.50	1.50	1.30	1.43	1.50	1.40	1.79	1.68	1.80	1.80
	Area/%	30.9	15.3	9.7	25.0	19.1	80.5	19.5	61.8	38.2	90.6	9.4
**F_{16}CuPc**	BE/eV	287.3	286.4	/	285.2	/	399.1	/	687.6	/	935.7	/
	FWHM	1.12	1.20	/	1.15	/	1.24	/	1.50	/	1.61	/
	Area/%	49.6	22.8	/	27.6	/	100.0	/	100.0	/	100.0	/

Table S5 Detailed peak fitting parameters in C 1s, F 1s, N 1s and Cu $2p_{3/2}$ XPS core-level spectra for Na with increasing $F_{16}CuPc$ deposition.

**Na/F_{16}CuPc**		**C 1s**					**N 1s**		**F 1s**		**Cu 2p**	
		**C_F**	**C_N**	**$C_{N\text{-}Na}$**	**C_C**	**$C_{F\text{-}Na}$**	**N_C**	**N_{Na}**	**F_C**	**F_{Na}**	**Cu(II)**	**Cu(I)**
**Na**	BE/eV	/	/	/	/	/	/	/	/	/	/	/
	FWHM	/	/	/	/	/	/	/	/	/	/	/
	Area/%	/	/	/	/	/	/	/	/	/	/	/

**0.12 nm**	BE/eV	/	/	287.3	286.7	285.9	400.2	399.5	/	686.4	/	935.0
	FWHM	/	/	1.40	1.60	1.60	1.31	1.30	/	1.46	/	1.80
	Area/%	/	/	23.3	25.6	51.1	31.3	68.7	/	100.0	/	100.0
**0.3 nm**	BE/eV	/	/	287.3	286.7	285.9	399.9	399.3	/	686.3	936.6	934.8
	FWHM	/	/	1.33	1.20	1.40	1.31	1.30	/	1.40	1.70	1.60
	Area/%	/	/	23.3	25.6	51.1	42.7	57.3	/	100.0	27.5	72.5
**0.6 nm**	BE/eV	288.1	/	286.7	286.0	285.1	399.7	399.1	688.9	685.7	936.3	934.5
	FWHM	1.60	/	1.31	1.40	1.40	1.31	1.30	1.70	1.58	1.70	1.80
	Area/%	15.6	/	25.8	28.3	30.3	46.5	53.5	43.0	57.0	66.2	33.8
**1.0 nm**	BE/eV	288.0	/	286.4	285.7	285.1	399.7	399.0	688.9	685.4	936.0	934.2
	FWHM	1.60	/	1.50	1.40	1.30	1.29	1.22	1.69	1.49	1.63	1.30
	Area/%	38.7	/	25.1	27.6	8.6	46.5	53.5	83.4	16.6	95.0	5.0
**1.8 nm**	BE/eV	287.8	/	286.3	285.7	/	399.6	399.0	688.6	/	935.9	/
	FWHM	1.70	/	1.70	1.40	/	1.25	1.22	1.61	/	1.59	/
	Area/%	51.1	/	23.3	25.6	/	46.9	53.1	100.0	/	100.0	/

References

1. Schwieger, T.; Peisert, H.; Golden, M. S.; Knupfer, M.; Fink, J., Electronic structure of the organic semiconductor copper phthalocyanine and K-CuPc studied using photoemission spectroscopy. *Physical Review B* **2002,** *66* (15).

2. Ding, H. J.; Gao, Y., Modification on the Electronic Structure of Organic Semiconductor by Alkali Metal. *ECS Transactions* **2008,** *11*, 1.

3. Kafafi, Z. H.; Watkins, N. J.; Antoniadis, H.; Yan, L.; Zorba, S.; Gao, Y.; Tang, C. W., Evidence of electron and hole transfer in metal/CuPc interfaces. In *Organic Light-Emitting Materials and Devices VI*, 2003.

4. Shen, C.; Kahn, A., Electronic structure, diffusion, and p-doping at the Au/F_{16}CuPc interface. *Journal of Applied Physics* **2001,** *90* (9), 4549-4554.

5. Peisert, H.; Knupfer, M.; Schwieger, T.; Fuentes, G. G.; Olligs, D.; Fink, J.; Schmidt, T., Fluorination of copper phthalocyanines: Electronic structure and interface properties. *Journal of Applied Physics* **2003,** *93* (12), 9683-9692.

6. Shen; Chongfei; Kahn; Antoine; Schwartz; Jeffrey, Role of metal-molecule chemistry and interdiffusion on the electrical properties of an organic interface: The Al-F_{16}CuPc case. *Journal of*

Applied Physics **2001**.

7. Shima, M.; Tsutsumi, K.; Tanaka, A.; Onodera, H.; Tanemura, M., Chemical state analysis using Auger parameters for XPS spectrum curve fitted with standard Auger spectra. *Surface and Interface Analysis* **2018,** *50* (11), 1187-1190.

8. Poulston, S.; Parlett, P. M.; Stone, P.; Bowker, M., Surface Oxidation and Reduction of CuO and Cu_2O Studied Using XPS and XAES. *Surface and Interface Analysis* **1996,** *24* (12), 811-820.

9. Tang, J.; Lee, C.; Lee, S., Chemical bonding and electronic structures at magnesium/copper phthalocyanine interfaces. *Applied Surface Science* **2006,** *252* (11), 3948-3952.

10. Yi, J.; Chen, J.; Yang, Z.; Dai, Y.; Li, W.; Cui, J.; Ciucci, F.; Lu, Z.; Yang, C., Facile Patterning of Laser-Induced Graphene with Tailored Li Nucleation Kinetics for Stable Lithium-Metal Batteries. *Advanced Energy Materials* **2019,** *9* (38), 1901796.

11. Yan, K.; Lu, Z.; Lee, H.-W.; Xiong, F.; Hsu, P.-C.; Li, Y.; Zhao, J.; Chu, S.; Cui, Y., Selective deposition and stable encapsulation of lithium through heterogeneous seeded growth. *Nature Energy* **2016,** *1* (3), 16010.
